# Device Performance During Simulated Emergency Infant Oropharyngeal Contamination: Effects of Airway Anatomy and Fluid Consistency

**DOI:** 10.3390/children13091149

**Published:** 2026-08-27

**Authors:** Ravi Jindal, Ryan Anderson, Manu Madhok, Todd DeFor, Kumar Belani

**Affiliations:** 1 Department of Anesthesiology, University of Minnesota, Minneapolis, MN 55455, USA; ravijindal1178@gmail.com; 2 Simulation Center, Children’s Minnesota, Minneapolis, MN 55404, USA; 3 Department of Emergency Medicine, Children’s Minnesota, Minneapolis, MN 55404, USA; 4 Clinical and Translational Science Institute, University of Minnesota, Minneapolis, MN 55455, USA

**Keywords:** airway contamination, aspiration, infant airway, oropharyngeal suction, simulation, suction catheter, Yankauer

## Abstract

**Highlights:**

**What are the main findings?**

- Suction performance differed between the open-container and infant-airway models.
- Although the Yankauer demonstrated superior intrinsic suction capability, anatomical constraints reduced the magnitude of these differences within the infant airway model.

**What are the implications of the main findings?**

- Data derived from unconstrained bench-top studies should not be directly extrapolated to pediatric airway management
- Findings support further evaluation of a complementary suction strategy using rigid suction for rapid bulk decontamination followed by flexible catheter suction for anatomically inaccessible regions.

**Abstract:**

**Background:** Sudden contamination of the pediatric oropharynx by regurgitated material, emesis, or blood is a time-critical airway emergency. Rapid clearance may be required to restore laryngeal visualization and permit ventilation and tracheal intubation. Comparative data for commonly available suction devices in anatomically constrained infant airways remain limited. We evaluated how airway anatomy and fluid consistency affect the performance of a rigid Yankauer suction tip and a 14 French (Fr) flexible suction catheter. **Methods:** This pilot simulation study used two complementary models: an anatomically constrained infant airway mannequin and an unconstrained open-container model. Water, whole milk, and plain yogurt (50 mL) represented contaminants of increasing consistency. Both devices were tested at a standardized wall-suction pressure of −200 mmHg, selected as a high-vacuum emergency bench condition rather than as a recommended pressure for routine pediatric suctioning. In the mannequin, suction time, evacuated pharyngeal volume, residual pharyngeal/esophageal volume, and recovered lung volume were measured. In the container model, time to complete evacuation was recorded. **Results:** In the infant airway mannequin, the Yankauer evacuated greater pharyngeal volumes for all three fluids. Suction time was shorter with the Yankauer for water but did not differ significantly for milk or yogurt. After yogurt contamination, residual pharyngeal/esophageal volume was greater with the Yankauer, while recovered lung volumes did not differ significantly between devices. In the unconstrained model, the Yankauer evacuated water and milk more rapidly and cleared all yogurt samples; the 14 Fr catheter did not completely evacuate yogurt within the five-minute limit in any trial. **Conclusions:** Under the standardized high-vacuum conditions of this simulation, relative device performance differed between the two experimental models and across fluids of differing consistency. The Yankauer provided greater bulk removal, whereas the flexible catheter left less residual yogurt in the anatomically constrained model. These bench findings do not establish the safety or clinical superiority of either device at recommended pediatric suction pressures and support further evaluation at lower pressures and during simulated laryngoscopy.

## 1. Introduction

Airway contamination caused by regurgitation, emesis, blood, or aspirated material is a time-critical problem during pediatric airway management. Copious material can obscure the glottis, impede ventilation, delay tracheal intubation, and contribute to hypoxemia and pulmonary aspiration. These consequences may develop particularly rapidly in infants because their smaller airway dimensions, higher oxygen consumption, reduced functional residual capacity, and limited physiologic reserve shorten the safe apneic period [1]. For this bench comparison, suction was standardized at −200 mmHg to reproduce an extreme high-vacuum emergency condition in which wall suction may be maximized during sudden, large-volume contamination. This setting exceeds routine recommended pediatric suction pressures and should not be interpreted as clinical practice guidance.

Previous simulation studies have shown that suction performance is influenced by catheter diameter and lumen size. Nikolla et al. reported greater flow rates with large-bore suction systems than with a conventional Yankauer, and Finke et al. found that a DuCanto catheter evacuated fluids of differing viscosities more effectively than Yankauer and standard suction catheters [2,3]. Andreae et al. likewise reported improved evacuation of simulated emesis with alternative suction devices [4]. This work contributed to the development of Suction-Assisted Laryngoscopy Airway Decontamination (SALAD), which emphasizes early, continuous suction during management of a massively contaminated airway [5,6].

Most published studies, however, have evaluated devices in open containers, unconstrained bench systems, or adult airway models. Such experiments characterize intrinsic evacuation capability but do not reproduce the limited working space, restricted device maneuverability, or inaccessible dependent recesses of an infant oropharynx. It therefore remains uncertain whether performance in an unconstrained model predicts functional performance in an anatomically constrained infant airway.

The infant airway has a relatively large tongue, small oral cavity, short pharyngeal space, and more cephalad and anterior larynx compared with older children and adults [7,8]. These features may limit positioning of a rigid suction tip. A flexible catheter may reach confined or dependent regions more readily, despite its smaller lumen and lower intrinsic flow capacity. Thus, suction performance may reflect an interaction among device design, airway geometry, operator access, and contaminant properties.

We compared a standard Yankauer suction tip with a 14 Fr flexible suction catheter in two complementary models: an anatomically constrained infant airway mannequin and an unconstrained open container. We hypothesized that the Yankauer’s advantage under unconstrained conditions would be attenuated within the infant airway mannequin. The study was designed to compare devices under a standardized high-vacuum emergency simulation; it was not designed to determine a safe suction pressure for pediatric patients.

## 2. Methods

### 2.1. Study Design

This pilot bench simulation was conducted at the Children’s Minnesota Simulation Center. Two complementary experimental models were used. The open-container model evaluated intrinsic evacuation capability independent of airway anatomy, whereas the infant airway mannequin evaluated device performance within a confined upper airway. The project involved no patients, animals, or identifiable data and therefore did not require human-subjects review.

**Figure 1:**
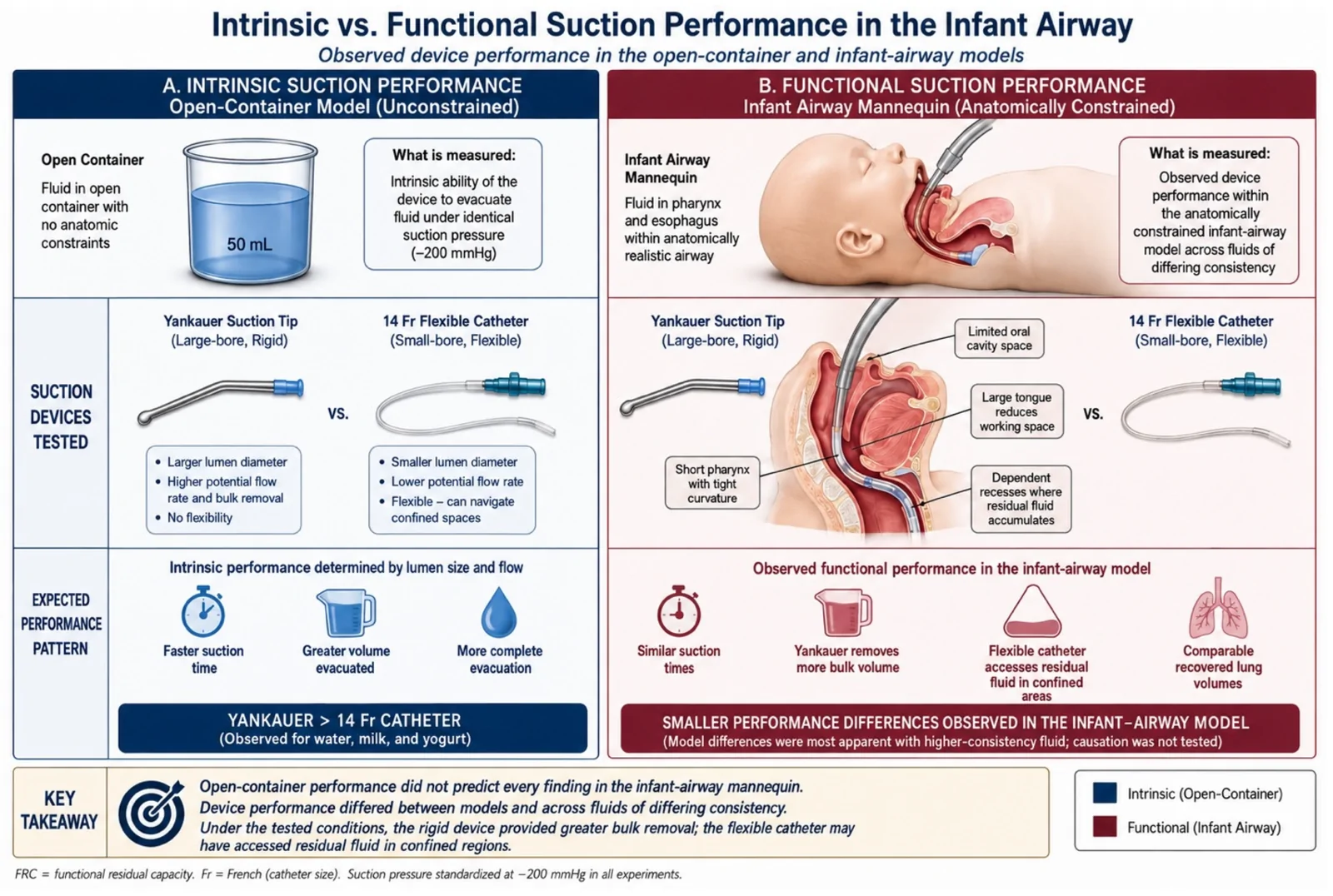
Conceptual comparison of device performance in the unconstrained open-container model and the anatomically constrained infant-airway mannequin. The open-container model assessed intrinsic evacuation capability under standardized suction conditions, whereas the mannequin model assessed device performance within a confined simulated airway. Device performance differed between the two models and across fluids of differing consistency. These observed differences may relate to device geometry, maneuverability, access to confined regions, fluid consistency, or simulator-specific characteristics; however, the study was not designed to establish a causal effect of airway anatomy. The proposed explanations are therefore hypothesis-generating and require further testing.

A conceptual comparison of the two experimental models and the factors that may influence device performance is provided in Figure 1.

### 2.2. Experimental Setup

**Figure 2:**
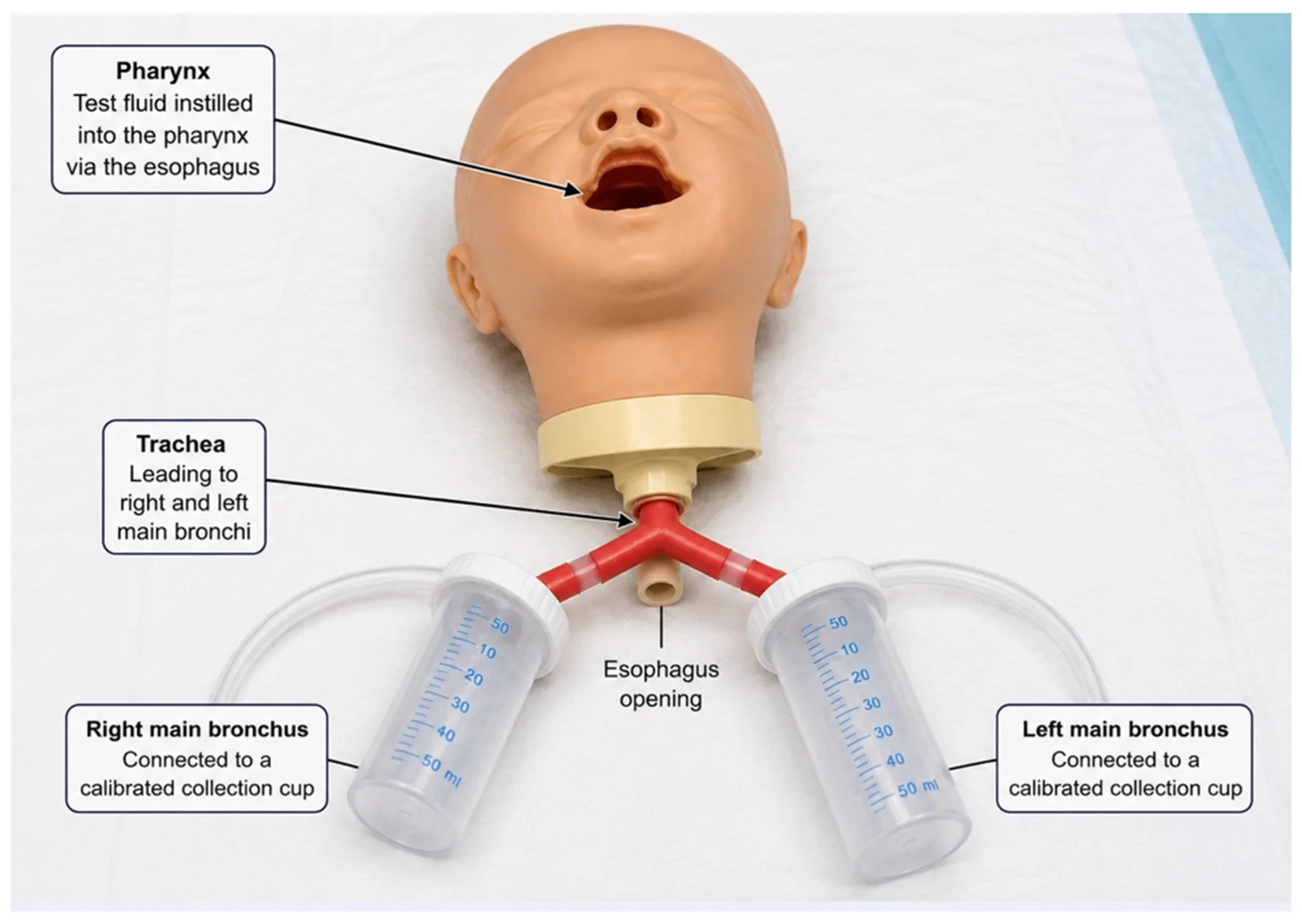
Infant-airway mannequin experimental setup. Test fluid was instilled into the pharynx through the esophageal opening. Calibrated collection cups connected to the right and left main bronchi were used to measure recovered lung volume. All experiments used a standardized wall-suction pressure of −200 mmHg.

An AirSim^®^ Baby X airway mannequin (TruCorp Ltd., Lurgan, Co. Armagh, Northern Ireland, UK; model TCJR10001X) was positioned supine (Figure 2). The simulator provides a confined infant upper-airway space and esophageal and bronchial outlets, permitting measurement of fluid distribution after simulated regurgitation. Although commercially described as anatomically realistic, it cannot reproduce living-tissue compliance, mucosal responses, airway reflexes, or dynamic airway collapse.

Wall suction was standardized at −200 mmHg throughout the experiments, using the same source, tubing, and collection system. Pressure was verified before each experimental session. This pressure was selected to represent a fixed high-vacuum emergency bench condition encountered when clinicians maximize wall suction during sudden, large-volume oropharyngeal contamination to restore visualization and facilitate airway control. It was not selected as a recommended setting for routine neonatal suctioning or suctioning through an endotracheal tube. Current neonatal resuscitation specifications generally use 80–100 mmHg, and the 2022 American Association for Respiratory Care guideline recommends keeping pressure below −120 mmHg during neonatal and pediatric artificial-airway suctioning [9,10]. Thus, the experimental setting intentionally addressed a different indication but exceeded pressures recommended for routine neonatal/pediatric suctioning.

Three reproducible test fluids represented progressively increasing consistency:

- Water (low consistency);
- Whole milk (intermediate consistency);
- Plain yogurt (high consistency).

The fluids were selected as pragmatic surrogates for increasingly thick airway contaminants. Their viscosity and other rheological properties were not formally measured; consequently, the terms low, intermediate, and high consistency are descriptive rather than quantitative [2,3,4].

### 2.3. Suction Devices

Two suction devices commonly available in pediatric operating rooms were evaluated:

- Yankauer suction tip (K87; Cardinal Health, Dublin, OH, USA);
- 14 French flexible suction catheter (T260C; Cardinal Health, Dublin, OH, USA).

The Yankauer was chosen as a commonly used rigid device for rapid bulk clearance of the oropharynx. The 14 Fr flexible catheter was chosen because it is readily available and can be directed into confined regions that may be difficult to reach with a rigid tip.

Identical suction tubing and collection canisters were used for all trials. One investigator performed every procedure with a standardized technique to reduce operator variability.

### 2.4. Infant Airway Mannequin Model

For each mannequin trial, 50 mL of test fluid was rapidly instilled through the esophageal opening into the pharyngeal cavity with a syringe. The syringe then remained attached to the esophageal port to limit retrograde drainage during suctioning.

Suction began immediately after instillation. Direct or video laryngoscopy and other airway adjuncts were not used. The operator passed the assigned device through all accessible regions of the pharynx. A trial ended when two complete passes yielded no additional fluid; this was the prespecified stopping criterion.

Each device–fluid combination was evaluated in five independent trials. Fluids were tested in the sequence water, whole milk, and yogurt. Between trials, the mannequin was emptied by suction through the esophageal port and then inverted; the pharyngeal cavity was visually inspected to confirm clearance before the next trial.

### 2.5. Outcome Measures

The following outcomes were recorded for each mannequin trial:

- Suction time (seconds);
- Pharyngeal volume evacuated (mL);
- Residual pharyngeal/esophageal volume (mL);
- Recovered lung volume (mL).

Suction time was measured from initiation of suction until the stopping criterion was reached. Evacuated pharyngeal volume was read directly from the calibrated suction collection canister. Recovered lung volume was read directly from the calibrated containers connected to the bronchial outlets. Residual pharyngeal/esophageal volume was not measured independently; it was calculated for each trial by mass balance as the 50 mL instilled volume minus the evacuated pharyngeal volume and recovered lung volume.

### 2.6. Unconstrained Open-Container Model

To characterize intrinsic performance without airway constraints, 50 mL of each fluid was placed in an open container and suctioned with each device at the same −200 mmHg pressure.

Each device–fluid combination was evaluated in three independent trials. Three repetitions were used because preliminary open-container trials showed little variability; five repetitions were retained for the mannequin model because catheter position and airway geometry introduced greater variability.

A trial was stopped at five minutes if complete evacuation had not occurred. This prespecified pragmatic upper limit was chosen to distinguish complete evacuation from functional failure while preventing indefinitely prolonged bench trials; 300 s also greatly exceeded the evacuation times observed for completed trials and the time frame relevant to emergency airway clearance. Accordingly, the yogurt trials involving the 14 Fr catheter represent unsuccessful, right-censored attempts rather than completed evacuation times.

### 2.7. Statistical Analysis

This exploratory pilot study emphasized effect magnitude, consistency, and uncertainty. No prospective sample-size calculation was performed.

Continuous outcomes are presented as individual observations, means, and 95% confidence intervals (CIs). Devices were compared separately within each fluid and model using two-sided independent-sample *t* tests. Mann–Whitney U tests were used as sensitivity analyses because normality could not be assessed reliably with the small sample sizes.

No adjustment was made for multiple comparisons; *p* values are therefore exploratory and should not be interpreted as confirmatory. A device-by-model interaction was not formally tested because the two models used different stopping rules, outcome structures, and numbers of repetitions. Differences between models are described as observed patterns rather than statistically established interactions.

The 14 Fr catheter did not completely evacuate yogurt in any open-container trial before the five-minute limit. These censored failures are reported descriptively and were not assigned an artificial completion time or included in a parametric comparison.

Statistical significance was defined as a two-sided *p*-value < 0.05. Interpretation emphasizes estimates and observed patterns rather than statistical significance alone.

## 3. Results

### 3.1. Suction Time in the Infant Airway Mannequin

**Figure 3:**
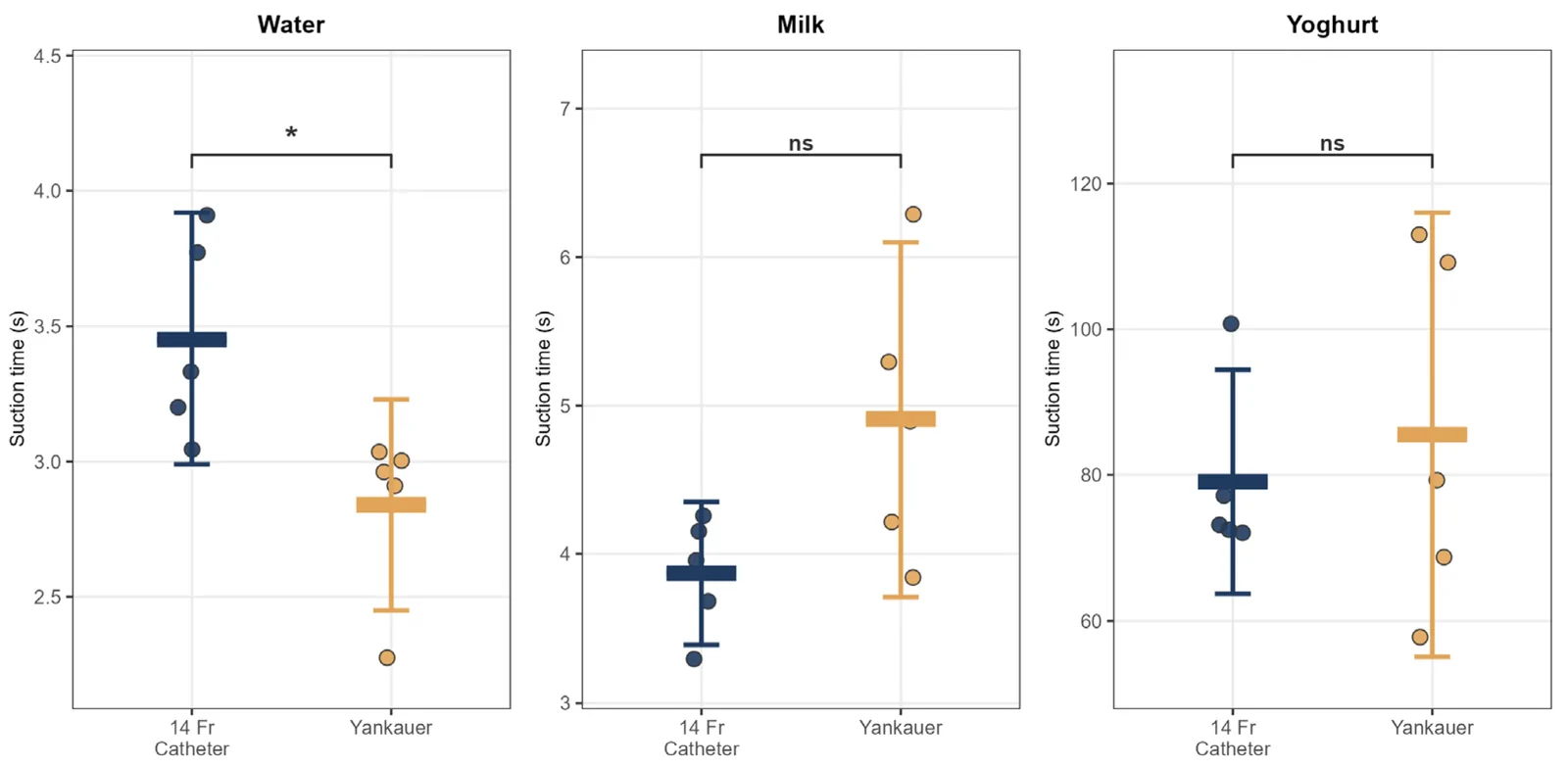
Suction time in the infant-airway mannequin model. Suction time for the Yankauer suction tip and 14 Fr flexible suction catheter during clearance of water, whole milk, and plain yogurt from the infant-airway mannequin. Points represent individual trials (*n* = 5 per device–fluid combination); horizontal bars indicate means, and error bars indicate 95% confidence intervals. Suction time was shorter with the Yankauer for water (*p* = 0.0221) but did not differ significantly between devices for milk (*p* = 0.0557) or yogurt (*p* = 0.6121). All trials used −200 mmHg wall suction. * *p* < 0.05; ns, not significant.

In the infant airway mannequin, the Yankauer evacuated water faster than the 14 Fr catheter (mean 2.84 s, 95% CI 2.45–3.23, versus 3.45 s, 95% CI 2.99–3.92; *p* = 0.0221) (Figure 3). Suction time did not differ significantly for whole milk (4.91 s, 95% CI 3.71–6.10, versus 3.87 s, 95% CI 3.39–4.35; *p* = 0.0557) or yogurt (85.55 s, 95% CI 55.09–116.01, versus 79.07 s, 95% CI 63.69–94.44; *p* = 0.6121). Suction time increased substantially with yogurt for both devices.

### 3.2. Pharyngeal Volume Evacuated

**Figure 4:**
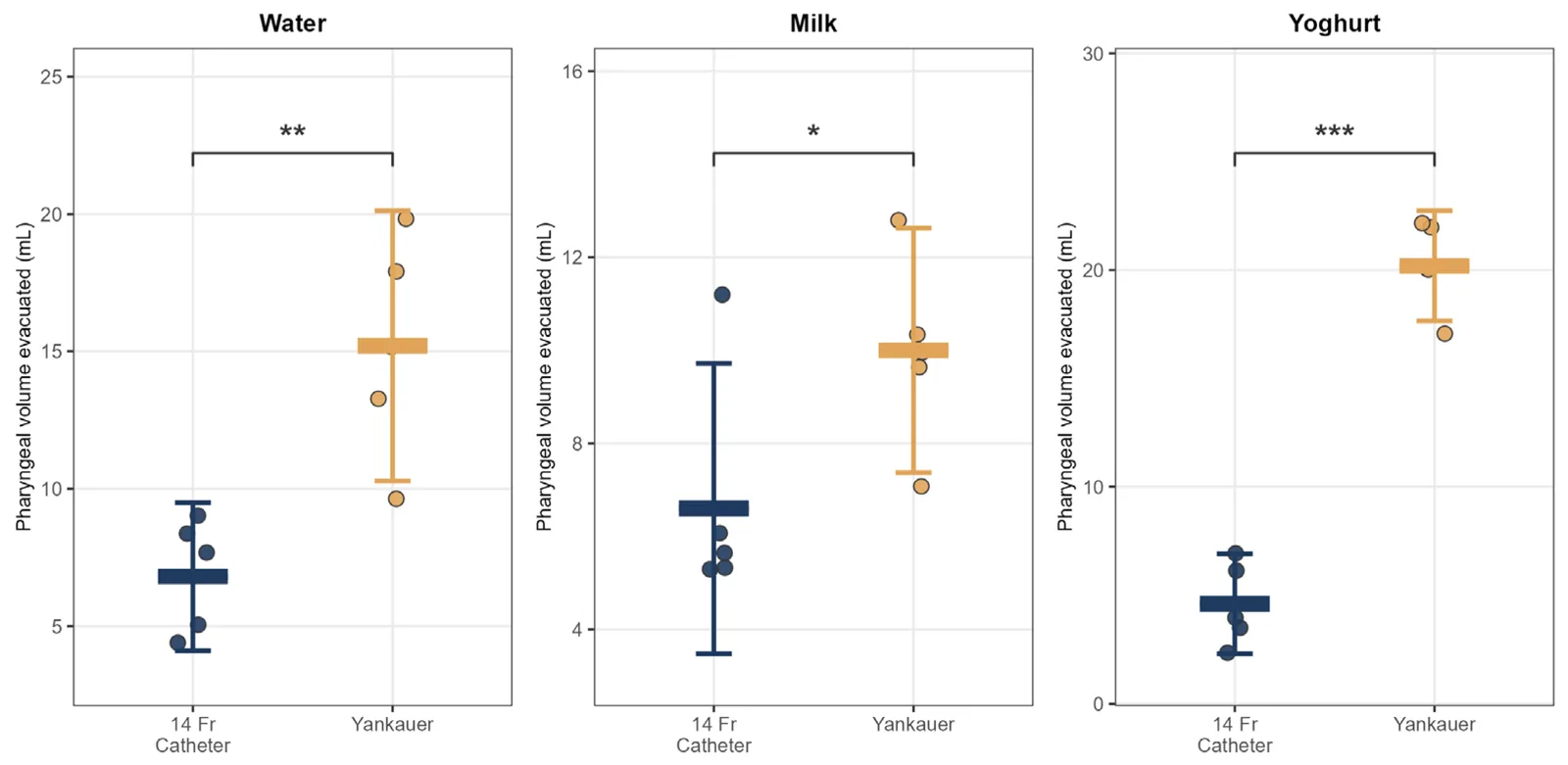
Pharyngeal volume evacuated in the infant-airway mannequin model. Pharyngeal volume evacuated by the Yankauer suction tip and 14 Fr flexible suction catheter during clearance of water, whole milk, and plain yogurt. Points represent individual trials (*n* = 5 per device–fluid combination); horizontal bars indicate means, and error bars indicate 95% confidence intervals. The Yankauer evacuated greater pharyngeal volumes for water (*p* = 0.0032), milk (*p* = 0.0494), and yogurt (*p* < 0.0001). All trials used −200 mmHg wall suction. * *p* < 0.05; ** *p* < 0.01; *** *p* < 0.001.

The Yankauer evacuated greater pharyngeal volumes for all three fluids (Figure 4). For yogurt, it removed 20.2 mL (95% CI 17.66–22.74) compared with 4.6 mL (95% CI 2.30–6.90) using the 14 Fr catheter (*p* < 0.0001). Differences were also observed for water (15.2 versus 6.8 mL; *p* = 0.0032) and whole milk (10.0 versus 6.6 mL; *p* = 0.0494).

### 3.3. Residual Pharyngeal/Esophageal Volume

**Figure 5:**
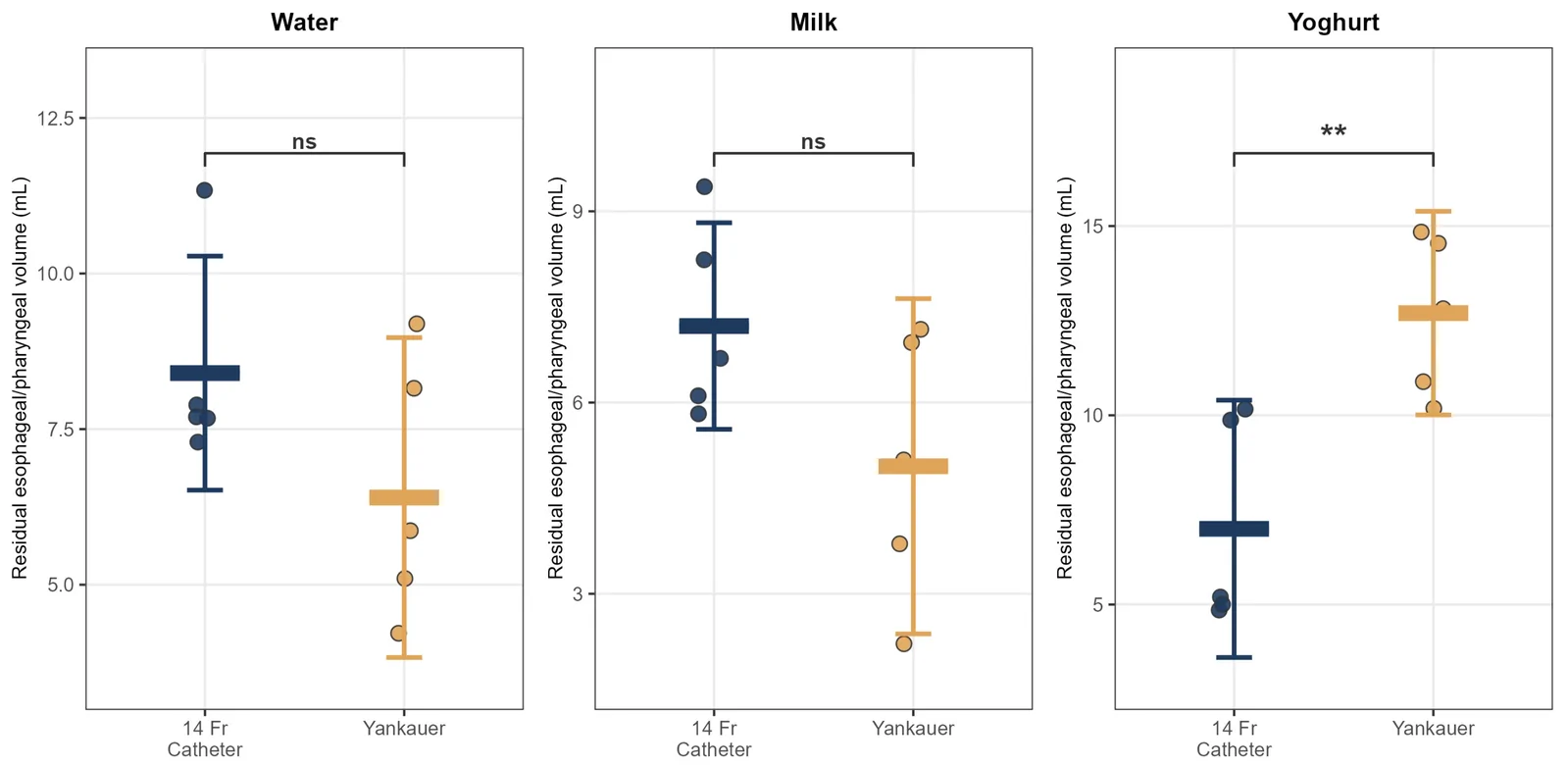
Residual pharyngeal/esophageal volume in the infant-airway mannequin model. Residual pharyngeal/esophageal volume after suctioning with the Yankauer suction tip or 14 Fr flexible suction catheter during clearance of water, whole milk, and plain yogurt. Points represent individual trials (*n* = 5 per device–fluid combination); horizontal bars indicate means, and error bars indicate 95% confidence intervals. Residual volume did not differ significantly between devices for water (*p* = 0.1199) or milk (*p* = 0.0836), but was greater with the Yankauer for yogurt (*p* = 0.0065). All trials used −200 mmHg wall suction. ** *p* < 0.01; ns, not significant.

Residual pharyngeal/esophageal volumes are shown in Figure 5. Differences were not statistically significant for water (Yankauer 6.4 versus catheter 8.4 mL; *p* = 0.1199) or whole milk (5.0 versus 7.2 mL; *p* = 0.0836). After yogurt contamination, residual volume was greater after Yankauer suction (12.7 versus 7.0 mL; *p* = 0.0065). This pattern is compatible with greater bulk removal through the Yankauer’s larger lumen but reduced access of its rigid tip to dependent recesses or areas around the tongue within the constrained mannequin. The flexible catheter may have conformed to the airway path and reached some of these regions more readily. Device position and regional fluid distribution were not directly measured, so this geometric explanation remains exploratory.

### 3.4. Recovered Lung Volume

**Figure 6:**
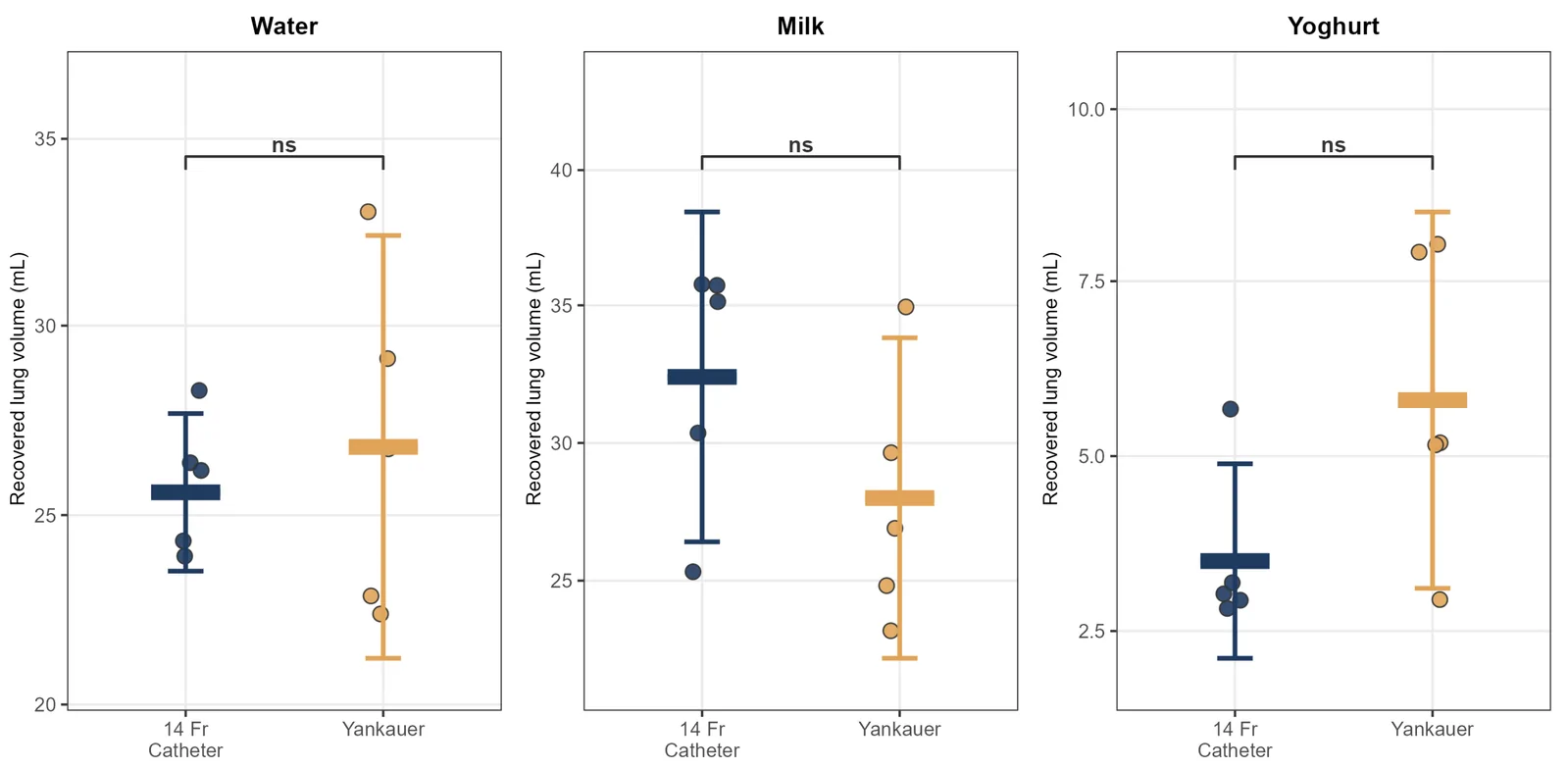
Recovered lung volume in the infant-airway mannequin model. Recovered lung volume after suctioning with the Yankauer suction tip or 14 Fr flexible suction catheter during clearance of water, whole milk, and plain yogurt. Points represent individual trials (*n* = 5 per device–fluid combination); horizontal bars indicate means, and error bars indicate 95% confidence intervals. No statistically significant between-device differences were observed for water (*p* = 0.5911), milk (*p* = 0.1819), or yogurt (*p* = 0.0680). All trials used −200 mmHg wall suction. ns, not significant.

Recovered lung volumes are shown in Figure 6. No statistically significant device differences were observed for water (Yankauer 26.8 versus catheter 25.6 mL; *p* = 0.5911), whole milk (28.0 versus 32.4 mL; *p* = 0.1819), or yogurt (5.8 versus 3.5 mL; *p* = 0.0680). These measurements describe the distribution of instilled fluid within the mannequin and should not be interpreted as clinical aspiration outcomes.

### 3.5. Unconstrained Open-Container Model

**Figure 7:**
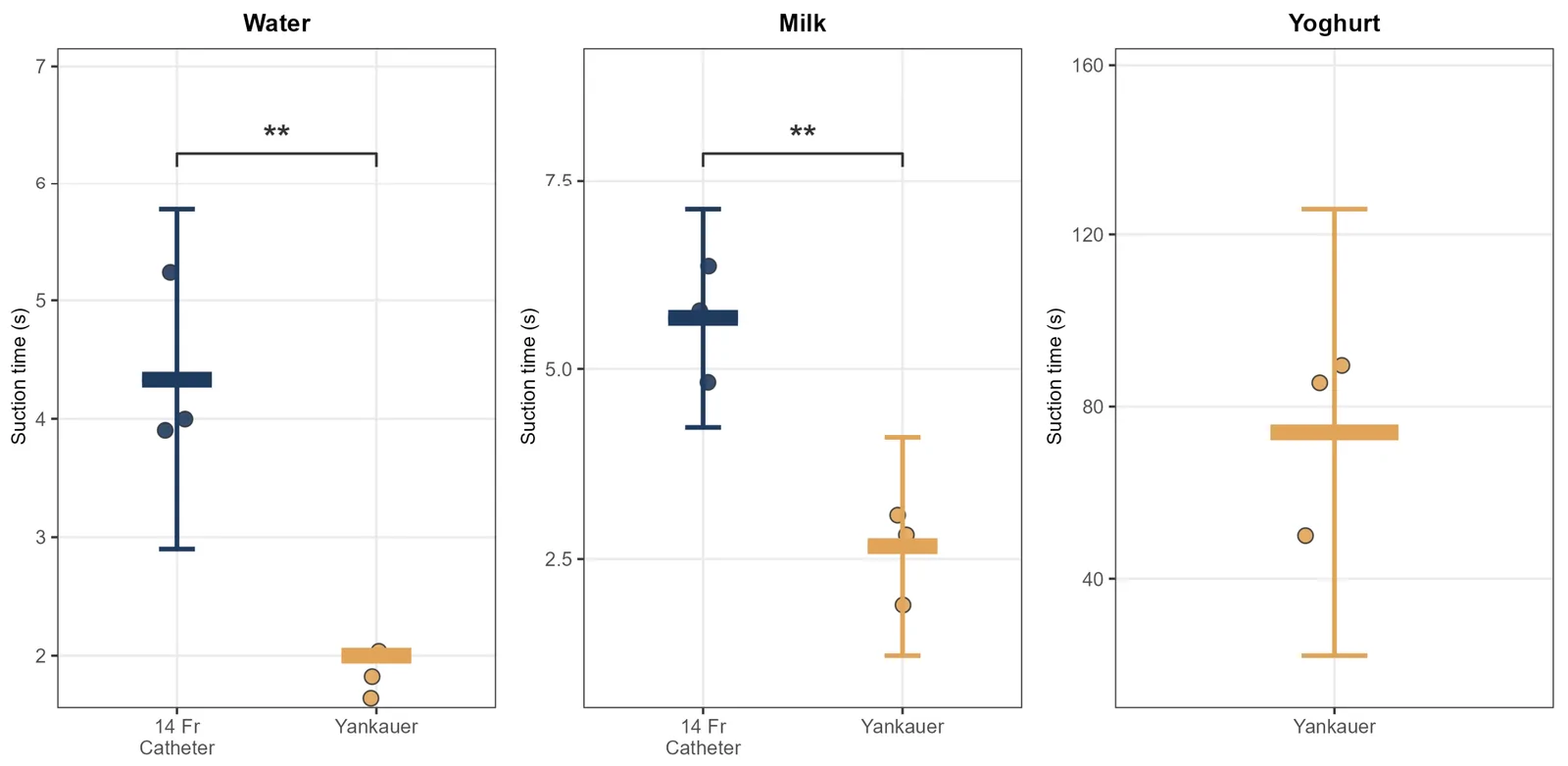
Suction time in the open-container model. Suction time for evacuation of water, whole milk, and plain yogurt with the Yankauer suction tip and 14 Fr flexible suction catheter in the open-container model. Points represent individual trials (*n* = 3 per device–fluid combination); horizontal bars indicate means, and error bars indicate 95% confidence intervals. The Yankauer evacuated water (*p* = 0.0022) and milk (*p* = 0.0031) faster than the 14 Fr catheter. For yogurt, the Yankauer completed evacuation, whereas the 14 Fr catheter did not complete evacuation within the prespecified 5 min limit in any trial; no formal between-device comparison was performed. All trials used −200 mmHg wall suction. ** *p* < 0.01.

In the open-container model, the Yankauer evacuated water faster than the 14 Fr catheter (2.00 s in every trial versus mean 4.33 s, 95% CI 2.90–5.77; *p* = 0.0022) and also evacuated whole milk faster (2.67 s, 95% CI 1.23–4.10, versus 5.67 s, 95% CI 4.23–7.10; *p* = 0.0031) (Figure 7). The Yankauer completely evacuated every yogurt sample (mean 74.0 s, 95% CI 22.13–125.87). The 14 Fr catheter did not completely evacuate yogurt within five minutes in any trial, so no formal time comparison was performed.

### 3.6. Summary of Findings

The open-container model showed a marked Yankauer advantage, especially for high-consistency material. In the infant mannequin, that advantage was less evident for suction time, although the Yankauer removed greater pharyngeal volumes. The flexible catheter left less residual yogurt. Because no formal model-by-device interaction was tested, these comparisons describe patterns and do not establish that airway anatomy caused the differences. The principal quantitative findings are summarized in Table 1.

## 4. Discussion

### 4.1. Principal Findings

This pilot simulation produced three principal findings. First, the Yankauer provided greater bulk evacuation under the tested high-vacuum conditions. Second, performance in an open container did not parallel every finding in the anatomically constrained infant mannequin: device suction times were similar for milk and yogurt, and the flexible catheter left less residual yogurt. Third, fluid consistency strongly influenced both devices, with yogurt producing prolonged suction and failure of the 14 Fr catheter to complete evacuation in the open container. Together, these findings show that device lumen, airway access, and contaminant consistency should be considered when interpreting bench suction performance.

### 4.2. Relationship to Previous Studies

Previous simulations have generally shown improved evacuation with larger suction lumens. Nikolla et al. reported higher flow rates with large-bore systems, Finke et al. demonstrated superior removal using the DuCanto catheter, and Andreae et al. reported improved evacuation of simulated emesis with alternative devices [2,3,4]. These studies informed the SALAD approach to the massively contaminated airway [5,6].

Our study extends this work by comparing the same devices in open-container and infant-mannequin settings. The results should not be taken as proof of an anatomical interaction because the models were not analyzed in a unified factorial design. They do, however, show that an unconstrained flow comparison alone may not capture access limitations within a small airway model.

### 4.3. Why the Infant Airway Behaved Differently

The Yankauer’s larger effective lumen favored rapid bulk removal in the open container. Within the mannequin, the smaller oral and pharyngeal spaces constrained device positioning, and fluid could enter dependent or less accessible regions. These conditions plausibly reduced the advantage of the rigid device.

Infant airway characteristics—including a relatively large tongue, limited oral space, short pharyngeal dimensions, and a cephalad larynx—can restrict maneuverability [7,8]. Commercial mannequins also differ from living airways in geometry, surface characteristics, compliance, and fluid–surface interactions. Therefore, the observed model differences may reflect both clinically relevant space constraints and simulator-specific properties.

The greater residual yogurt volume after Yankauer suction may indicate that the flexible catheter reached areas inaccessible to the rigid tip. This mechanism was not directly observed or measured, however, and alternative explanations include differences in catheter placement, wall adherence of the test fluid, and trial-to-trial fluid distribution.

### 4.4. Clinical Implications

The study represents an acute, high-volume oropharyngeal-contamination scenario rather than routine secretion management. During massive regurgitation or bleeding, the immediate goals are rapid bulk clearance, restoration of glottic visualization, and timely ventilation and intubation. Under the tested conditions, the Yankauer removed more pharyngeal material, but suction time was not consistently shorter in the mannequin and residual yogurt was greater.

These results do not establish a preferred clinical sequence. They instead suggest a hypothesis: a rigid device may be useful for accessible bulk material, while a flexible catheter may help reach residual material in confined regions. This complementary strategy requires direct testing during simulated laryngoscopy and ultimately clinical evaluation.

The use of negative pressure of −200 mmHg must also be interpreted carefully. It reflects a high-vacuum emergency bench condition used to compare devices, not a recommendation for routine pediatric suctioning. The study did not assess tissue injury or physiologic adverse effects, and it cannot determine whether the same relative performance would occur at 80–120 mmHg.

### 4.5. Implications for Pediatric SALAD

SALAD emphasizes proactive, continuous suction before and during laryngoscopy in a massively contaminated airway [5,6,11]. Most evidence derives from adult models, large-bore devices, and educational outcomes. The present findings caution against assuming that performance observed in an open container or adult airway will translate unchanged to an infant airway.

Future pediatric SALAD studies should evaluate rigid and flexible devices at clinically relevant pressure settings; incorporate direct or video laryngoscopy, ongoing contamination, and measures of glottic visualization and intubation success; and assess whether sequential or simultaneous suction strategies offer benefit.

### 4.6. Implications of Fluid Consistency

Water was cleared rapidly by both devices. With increasing consistency, suction took longer and the Yankauer’s bulk-evacuation advantage became more apparent. Yogurt produced the clearest separation in the open container but also exposed limitations of the rigid tip within the mannequin.

The selected fluids were reproducible but were not rheologically characterized and cannot reproduce the heterogeneous particulate composition, temperature, acidity, surface tension, or non-Newtonian behavior of actual gastric contents. The results therefore apply to the tested materials rather than to all clinical contaminants.

### 4.7. Study Limitations

This study has several important limitations. It was a small exploratory simulation using one commercial infant mannequin, one Yankauer design, one flexible catheter, and one operator. The mannequin cannot reproduce living-tissue compliance, mucosal injury, airway reflexes, bradycardia, dynamic collapse, or the physiologic consequences of aspiration. Commercial simulators may also differ materially and anatomically from human infant airways. Fluid viscosity was not formally measured, device order was not randomized, the operator could not be blinded, and the stopping criterion depended partly on operator assessment. The small numbers of trials limited assessment of distributional assumptions and statistical interactions, and multiple comparisons were not adjusted. The calculated residual volume may include measurement error and material adhering to internal surfaces. Most importantly, all trials used −200 mmHg, exceeding pressures recommended for routine neonatal/pediatric suctioning and artificial-airway suctioning [9,10]. Although this setting was chosen to model a high-vacuum emergency condition, the study did not evaluate pressure safety, and relative device performance at 80–120 mmHg is unknown. These findings are hypothesis-generating and should not be interpreted as clinical evidence supporting −200 mmHg in infants or children.

## 5. Conclusions

During simulated emergency infant oropharyngeal contamination at a standardized high-vacuum setting, the Yankauer removed greater bulk pharyngeal volumes, whereas the 14 Fr flexible catheter left less residual yogurt in the anatomically constrained model. Open-container performance did not predict every finding in the mannequin. These results do not establish pressure safety, clinical superiority, or a recommended pediatric suction strategy. Further studies should compare devices at clinically recommended pediatric pressures, use more anatomically and mechanically representative models, and incorporate laryngoscopy, airway visualization, and intubation outcomes.

## Figures and Tables

**Table 1:** Summary of suction performance in the infant airway mannequin and open-container models. Values are mean (95% confidence interval) unless otherwise indicated.

Model	Test Fluid	Device	*n*	Suction Time, s	Pharyngeal Volume Evacuated, mL	Residual Pharyngeal/ Esophageal Volume, mL	Recovered Lung Volume, mL	Between-Device *p* Values
Infant airway mannequin	Water	Yankauer	5	2.84 (2.45–3.23)	15.2 (10.28–20.12)	6.4 (3.83–8.97)	26.8 (21.22–32.38)	Time 0.0221; evacuated 0.0032; residual 0.1199; lung 0.5911
		14 Fr flexible catheter	5	3.45 (2.99–3.92)	6.8 (4.11–9.49)	8.4 (6.52–10.28)	25.6 (23.52–27.68)	
Infant airway mannequin	Whole milk	Yankauer	5	4.91 (3.71–6.10)	10.0 (7.37–12.63)	5.0 (2.37–7.63)	28.0 (22.18–33.82)	Time 0.0557; evacuated 0.0494; residual 0.0836; lung 0.1819
		14 Fr flexible catheter	5	3.87 (3.39–4.35)	6.6 (3.48–9.72)	7.2 (5.58–8.82)	32.4 (26.41–38.39)	
Infant airway mannequin	Plain yogurt	Yankauer	5	85.55 (55.09–116.01)	20.2 (17.66–22.74)	12.7 (10.01–15.39)	5.8 (3.11–8.49)	Time 0.6121; evacuated <0.0001; residual 0.0065; lung 0.0680
		14 Fr flexible catheter	5	79.07 (63.69–94.44)	4.6 (2.30–6.90)	7.0 (3.60–10.40)	3.5 (2.11–4.89)	
Open container	Water	Yankauer	3	2.00 in all trials	50 (complete evacuation)	—	—	Time 0.0022
		14 Fr flexible catheter	3	4.33 (2.90–5.77)	50 (complete evacuation)	—	—	
Open container	Whole milk	Yankauer	3	2.67 (1.23–4.10)	50 (complete evacuation)	—	—	Time 0.0031
		14 Fr flexible catheter	3	5.67 (4.23–7.10)	50 (complete evacuation)	—	—	
Open container	Plain yogurt	Yankauer	3	74.00 (22.13–125.87)	50 (complete evacuation)	—	—	Not tested
		14 Fr flexible catheter	3	>300; incomplete in all trials	Incomplete within 5 min	—	—	

Abbreviations: Fr, French. Dashes indicate outcomes not measured in the open-container model. The 14 Fr catheter did not completely evacuate yogurt within the predefined 5 min limit in any open-container trial; therefore, no formal between-device comparison was performed for that condition. Because the study was exploratory and involved small numbers of repeated trials, emphasis should be placed on effect size, consistency of findings, and 95% CIs rather than *p* values alone. All experiments used a standardized suction pressure of −200 mmHg to model a high-vacuum emergency bench condition. The study did not evaluate pressure safety, and the findings should not be interpreted as supporting routine use of −200 mmHg in pediatric patients.

## Data Availability

The original contributions presented in this study are included in the article. Further inquiries can be directed to the corresponding author.
